# Repetitive finger movement and circle drawing in persons with Parkinson’s disease

**DOI:** 10.1371/journal.pone.0222862

**Published:** 2019-09-23

**Authors:** Elizabeth L. Stegemöller, Andrew Zaman, Jennifer Uzochukwu

**Affiliations:** 1 Department of Kinesiology, Iowa State University, Ames, Iowa, United States of America; 2 Department of Physical Therapy, University of Alabama at Birmingham, Birmingham, Alabama, United States of America; Emory University, UNITED STATES

## Abstract

Little is known regarding how repetitive finger movement performance impacts other fine motor control tasks, such as circle drawing, in persons with Parkinson’s disease (PD). Previous research has shown that impairments in repetitive finger movements emerge at rates near to and above 2 Hz in most persons with PD. Thus, the purpose of this study was to compare circle drawing performance in persons with PD that demonstrate impairment in repetitive finger movement and those that do not. Twenty-two participants with PD and twelve healthy older adults completed the study. Only participants with PD completed the repetitive finger movement task. From the kinematic data for the repetitive finger movement task, participants were grouped into Hasteners and Non-Hasteners. Participants with PD and the healthy older adults completed a series of circle drawing tasks at two different target sizes (1 cm and 2 cm) and three pacing conditions (Self-paced, 1.25 Hz, and 2.5 Hz). Kinematic and electromyography data were recorded and compared between groups. Results revealed that, in general, persons with PD demonstrate impairments in circle drawing and associated electromyography activity compared to healthy older adults. Moreover, persons with PD that hasten during repetitive finger movements demonstrate significantly increased movement rate during circle drawing, while those persons with PD that do not hasten demonstrate a significant increase in width variability. This suggests that differing motor control mechanisms may play a role in the performance of fine motor tasks in persons with PD. Continued research is needed to better understand differences in circle drawing performance among persons with PD to inform future development of patient-centered treatments.

## Introduction

Research has shown that persons with Parkinson’s disease (PD) demonstrate impairments in fine motor tasks, such as writing tasks. Handwriting and circle drawing are small, slow, and often illegible [1–5]. These changes in writing may be a consequence of other PD symptoms such as tremor, rigidity, or bradykinesia, slowness of movement. However, bradykinesia may have the greatest impact on functional disability in persons with PD [6,7]. While repetitive movements, such as finger tapping, are used in the clinical assessment of bradykinesia, there is no assessment of other fine motor tasks on the motor Unified Parkinson’s Disease Rating Scale (UPDRS, part 3). Patients are asked about fine motor tasks in other portions of the UPDRS (i.e. part 2). Thus, subjective report from the patient combined with observed repetitive finger movement by the neurologist often results in the limited assessment of fine motor tasks. However, little is known regarding how repetitive movement performance impacts writing tasks, such as circle drawing, in persons with PD. A better understanding of how repetitive movements impact fine motor tasks may improve the assessment of persons with PD.

Previous research from our group has shown that impairments in simple repetitive finger movements emerge at rates near to and above 2 Hz in most persons with PD, and this impairment is not improved with medication [8,9]. Moreover, research from our group has shown that impairments in repetitive finger movements are associated with a decline in performance on the Purdue pegboard assembly task and buttoning, as well as quality of life [10,11]. Taken together, this may suggest that impairments in repetitive finger movement may also impact other fine motor movements that impact quality of life, such as circle drawing. Thus, the purpose of this study was to compare circle drawing performance in persons with PD that demonstrate impairment in repetitive finger movement and those who do not.

Writing performance often becomes worse when persons with PD are asked to write with increasing demands, such as larger or faster than normal [5,12, 13]. Moreover, changes in muscle activity may contribute to small and slow handwriting in persons with PD [5,14,15]. Thus, in this study kinematic and electromyography (EMG) data were collected while persons with PD and healthy older adults (HOAs) drew circles at a small (1 cm) and large (2 cm) size and at a self-paced, slow paced (1.25 Hz), and fast paced (2.5 Hz) rate. Given that impairments in repetitive finger movements, which are characterized by an increase in movement rate and a decrease in movement amplitude, emerge at pacing rates near to and above 2 Hz [8,9], we hypothesize that participants with PD that demonstrate impairments in repetitive finger movement will perform worse 1) when writing the small circles and 2) when writing at the faster pacing rate when compared to healthy older adults.

## Methods

### Participants

Twenty-two participants diagnosed with idiopathic PD (mean age 68 ± 11 years; 9/22 male, and 20/22 right handed) and 12 HOAs (mean age 71 ± 9 years; 2/12 male; 12/12 right handed) completed the study. Participants with PD reported an average disease duration of 8.3 ± 5.4 years and 50% of the participants reported that the right side was their more affected side. All participants with were tested 1 to 1.5 hours after taking their regular Parkinsonian medication. All participants gave their written informed consent prior to inclusion in the study, and the Iowa State University Institutional Review Board approved the procedures.

### Procedure

A standard diameter ballpoint pen and two sheets of lined paper were provided. One sheet of paper had lines spaced 1cm apart, and the other had lines spaced 2cm apart. Participants were instructed to draw repetitive circles clockwise, without lifting the pen, on the sheets of paper matching the size of the lines with their dominant hand. The circles were drawn over one another so that there was no lateral movement of the pen. For both sizes, participants were asked to draw the circles in time with a pacing tone that was presented at either 1.25 Hz or 2.5 Hz, in which the apex of the circle was synchronized to each tone. Participants were also asked to draw circles at their self-selected pace with no pacing tone. Thus, there were a total of six conditions were completed: 1) small (1cm)/self-paced, 2) small/1.25 Hz, 3) small/2.5 Hz, 4) large (2 cm)/self-paced, 5) large/1.25 Hz, and 6) large/2.5 Hz. Participants completed 20 circles per condition, and the order of each condition was randomized among participants. Pen kinematics were recorded at 200 Hz from an electromagnetic sensor (Ascension), placed near the tip of the ballpoint pen. Bipolar EMG sensors (Delsys) were placed on the index finger extensor (extensor digitorum communis (EDC)), and flexor (first dorsal interosseous (FDI)) of the hand used to write.

**Fig 1:**
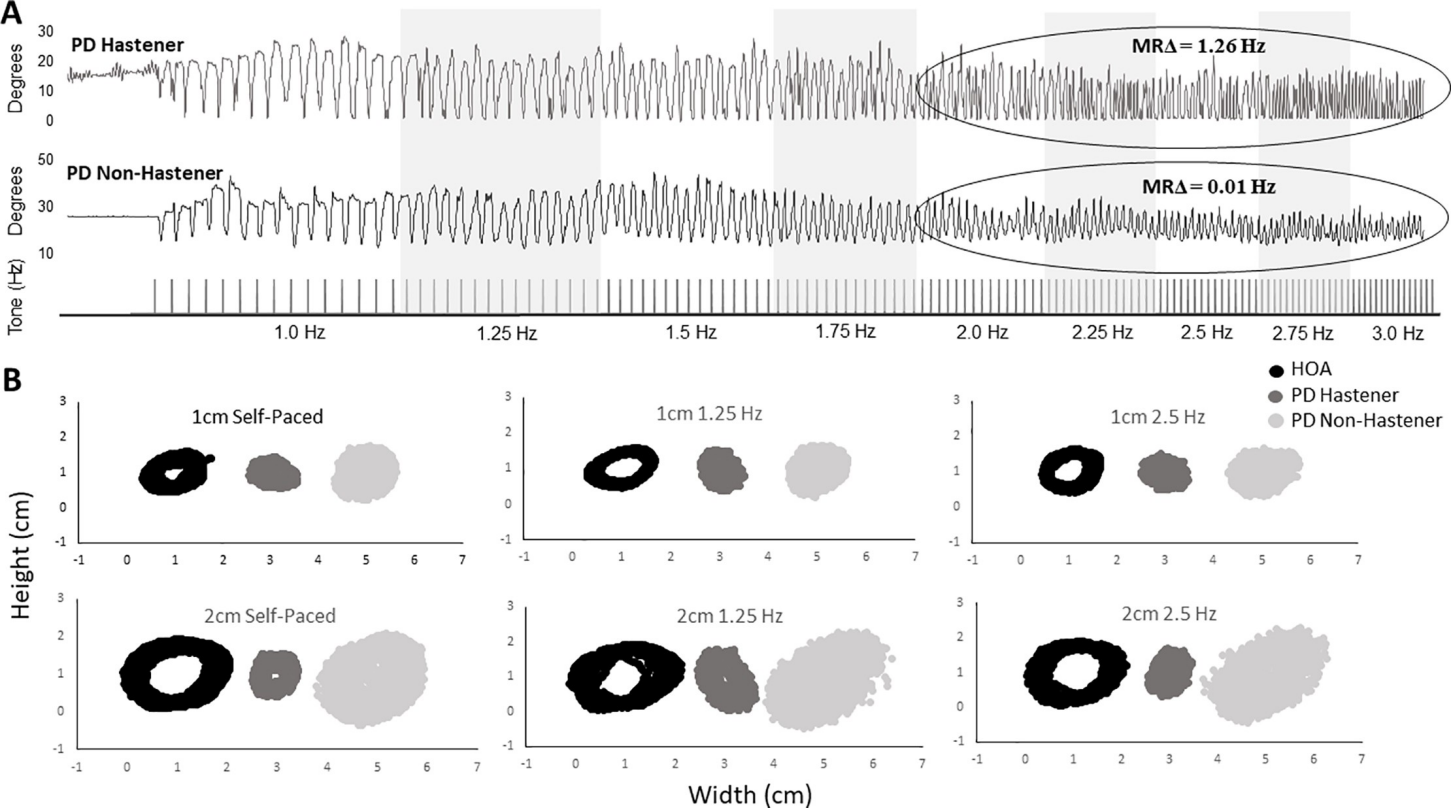
Individual examples of repetitive finger movement performance (A) and circle drawing (B) for one participant with PD that hastens and one participant with PD that does not hasten.

Participants with PD also completed an unconstrained repetitive finger movement task using their most affected hand. A pacing tone that began at a rate of 1.0 Hz and incremented by 0.25 Hz until reaching 3.0 Hz with fifteen tones at each rate was presented (Fig 1). Participants were asked to tap their index finger in time with the pacing tone while the arm and hand was secured in the pronated position. Three trials were collected. This task has been used previously to assess impairments in repetitive finger movements at rates near to and above 2 Hz in persons with PD [8,9]. Finger movement was collected using a goniometer, data acquisition board (Micro 1401, Cambridge Electronic Design, UK), and software (Spike2, CED). Signals were digitized at a sampling rate of 100Hz.

### Data analysis

For the circle drawing task, circle height, circle width, and movement rate were obtained. A digital representation of the circle drawing was reproduced from the pen sensor data using a project-specific Java program. Each time the individual reached their apex in the circle signified the start of a new circle. Circle height was determined as the anterior to posterior diameter of the circle, and circle width was determined as the medial to lateral diameter of the circle. Movement rate was calculated as the time from reaching the apex of one circle to reaching the apex of the next circle. Mean and standard error values were obtained for each kinematic outcome measure for each condition. The coefficient of variation (CV), standard deviation divided by the mean, was also calculated for each outcome measure for each condition.

Peak EMG and EMG area under the curve were obtain for both the EDC and FDI muscles. The root mean square was calculated for the EMG signal and filtered with a 20 to 500 Hz band-pass filter, and a 59-61Hz notch filter. Peak EMG amplitude was calculated by averaging the peak EMG amplitude for each written circle and averaged for each condition. EMG area under the curve was calculated by adding the trapezoidal area between successive points for the duration of the trial and averaged for each condition. The CV was also calculated for each EMG outcome measure for each condition.

Only participants with PD completed the repetitive finger movement task. For this task, peak flexion was obtained for each movement and peak-amplitude and movement rate difference (MRΔ) were determined. Peak-amplitude was normalized to the amplitude at 1.0 Hz. MRΔ was calculated as the difference between the actual movement rate and tone rate. For each participant with PD, peak amplitude and MRΔ was averaged across all tone rates less than 2.0 Hz and for all tone rates of 2.0 Hz and greater, returning two values of peak amplitude and MRΔ for each participant with PD. From this data, participants with PD were categorized into two groups based on their movement rate when the pacing tone exceeded 2 Hz or above, Hasteners (n = 9) and Non-Hasteners (n = 13). For the entire sample (n = 22), the standard deviation for MRΔ at 1 Hz was calculated. Participants that moved faster than two standard deviations for at least 3 consecutive tone rates at 2 Hz or above were categorized into the Hasteners group [9]. All other participants were categorized into the Non-Hastener group. See Table 1 for demographics and repetitive finger movement data for each PD group.

**Table 1:** Demographics and repetitive finger movement data for each group.

	Age (years)	Gender (% male)	Handedness (% right handed)	MAS (% dominant)	Peak Amp < 2.0 Hz	Peak Amp > 2.0 Hz	MRΔ < 2.0 Hz	MRΔ > 2.0 Hz
**Hasteners**	68 ± 4	67	100	22	1.04 ± 0.04	0.95 ± 0.09	0.21 ± 0.10	0.79 ± 0.17
**Non-Hasteners**	68 ± 3	23	85	54	0.96 ± 0.05	0.89 ± 0.10	0.04 ± 0.01	0.02 ± 0.04
***p* value**	0.96	0.04*	0.22	0.14	NA	NA	0.06	<0.001*

MRΔ = Movement Rate Difference; Amp = Amplitude; NA = Not Applicable; MAS = Most Affected Side. Mean and standard error are shown.

Asterisks (*) designate differences between Hastener and Non-Hastener groups.

Independent t-tests were not completed for Peak Amp as no group effects or interaction effects were revealed.

### Statistical analysis

All data was normally distributed. To determine if there were differences between PD groups, independent t-tests were completed for age, and chi square tests were completed for gender, handedness, and most affected side. To determine if there were differences between repetitive finger movement performance, a 2x2 repeated measures analysis of variance (ANOVA) was completed for both peak amplitude and MRΔ. The within group factor was pacing rate (< 2.0 Hz vs. > 2.0 Hz), and the between group factor was group (Hasteners vs. Non-Hasteners). Post hoc analysis was completed using independent t-tests with Bonferroni correction.

To determine the effects of circle size on movement rate and movement rate CV, separate 2 (1 cm vs. 2 cm) x 3 (Hasteners vs. Non-Hasteners vs. HOAs) repeated measure ANOVAs were completed for the self-paced, 1.25 Hz, and 2.5 Hz conditions. To determine the effects of pacing rate on circle height and circle height CV, separate 3 (self-paced vs.1.25 Hz vs. 2.5 Hz) x 3 (Hasteners vs. Non-Hasteners vs. HOAs) repeated measure ANOVAs were completed for small (1 cm) and large (2 cm) circles. To determine the effects of pacing rate on circle width and circle width CV, separate 3 (self-paced vs.1.25 Hz vs. 2.5 Hz) x 3 (Hasteners vs. Non-Hasteners vs. HOAs) repeated measure ANOVAs were also completed for small and large circles. Post hoc analysis was completed using Tukey’s Honestly Significant Difference test when a main effect of group or interaction effect was revealed.

Similarly, to determine the effects of circle size on EMG activity and EMG CV between Hasteners and Non-Hasteners, separate 2 (1 cm vs. 2 cm) x 3 (Hasteners vs. Non-Hasteners vs. HOAs) repeated measure ANOVAs were completed for the self-paced, 1.25 Hz, and 2.5 Hz conditions for each outcome measure (peak EDC, peak FDI, EDC area, and FDI area). To determine the effects of pacing rate on EMG activity and EMG CV, separate 3 (self-paced vs.1.25 Hz vs. 2.5 Hz) x 3 (Hasteners vs. Non-Hasteners vs. HOAs) repeated measure ANOVAs were also completed for small and large circles for each outcome measure. Post hoc analysis was completed using Tukey’s Honestly Significant Difference test if a main effect of group or interaction effect was revealed. If a main effect of pacing rate was revealed, post hoc analysis was completed using paired t-tests with Bonferroni correction.

## Results

Table 1 shows comparisons between groups for age, gender, handedness, and most affected side. Results revealed a significant difference in gender (*p* = 0.04), indicating a higher percentage of males in the hastener group. For the most affected side, analysis was completed to determine if the number of participants with their dominant hand as their most affected side was different between groups. Given that participants were using their dominant hand to write, whether it was the most affected side may influence results. However, hastener and non-hastener groups did not differ in which side of the body was more affected by PD (*p* = 0.15).

Fig 1A shows a representative sample of repetitive finger tapping performance for one participant in the Hastener group and one participant in the Non-Hastener group. The MRΔ at rates above 2 Hz was much higher in the participant who hastened compared to the participant that did not. Table 1 shows group kinematic data from the repetitive finger movement task. For peak amplitude, results revealed no main effect of pacing rate (*F*(1) = 1.37, *p* = 0.25), no main effect of group (*F*(1) = 0.056, *p* = 0.46), and no interaction effect (*F*(1) = 0.03, *p* = 0.87). In contrast, for MRΔ, results revealed a main effect of pacing rate (*F*(1) = 8.57, *p* = 0.008), a main effect of group (*F*(1) = 39.84, *p* < 0.001), and an interaction effect (*F*(1) = 10.34, *p* = 0.004). Confirming the grouping of PD participants, post hoc analysis revealed significant differences in MRΔ between the Hastener and Non-Hastener groups at pacing rates above 2.0 Hz (*t*(20) = 5.20, *p* < 0.001), but not at pacing rate below 2.0 Hz (*t*(20) = 1.20, *p* = 0.06). This indicates that the hasteners moved faster at rates above 2.0 Hz than the non-hasteners.

Fig 1B shows a representative sample of circle drawing performance for one participant in the Hastener group, one participant in the Non-Hastener group, and one HOA for each pacing rate and each circle size. In general, the PD Hastener produces smaller circles than both the PD Non-Hastener and HOA across all conditions. However, the PD Non-Hastener group demonstrated more variable performance, especially for the larger circles.

**Fig 2:**
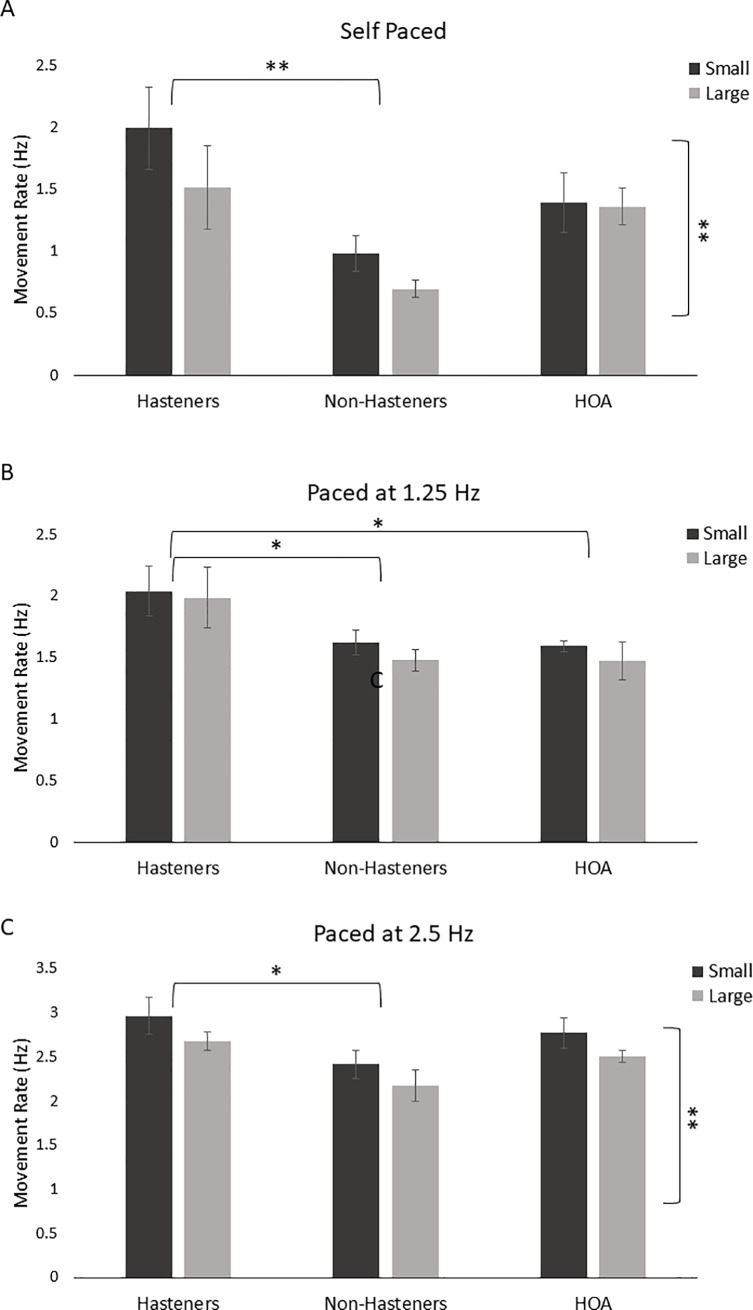
Movement rate for the PD Hastener, PD Non-Hastener, and HOA groups for self-paced (A), paced at 1.25 Hz (B), and paced at 2.5 Hz (C) conditions for both small and large circle sizes. The dotted line represents the intended pace. Horizontal brackets represent significant differences between groups. Vertical brackets represent a significant effect of circle size. *p < 0.05, **p < 0.01.

Fig 2 shows the effects of circle size on movement rate among groups. Overall, the Hastener group moved faster than the Non-Hastener group for both circle sizes and across all pacing rates. For circle size (1 cm vs. 2 cm), all groups tended to move faster when writing the smaller circle. Statistical results revealed a significant effect of size (*F*(1) = 8.452, *p* = 0.007) and group (*F*(2) = 5.598, *p* = 0.009), but no interaction effect (*F*(2) = 1.321, *p* = 0.282) for the self-paced condition (Fig 1A). For the pacing rate of 1.25 Hz, a main effect of group (*F*(1) = 3.953, *p* = 0.030) was revealed, but there was no significant difference for circle size (*F*(2) = 2.179, *p* = 0.151) and no interaction effect (*F*(2) = 0.144, *p* = 0.867) (Fig 1B). For the pacing rate of 2.5 Hz, results revealed a main effect of size (*F*(1) = 7.923, *p* = 0.009) and a main effect of group (*F*(2) = 3.764, *p* = 0.035). There was no interaction effect (*F*(2) = 0.017, *p* = 0.983) (Fig 1C). Post hoc comparisons between groups revealed significant differences between the Hastener group and Non-Hastener group for the self-paced (*p* = 0.007), 1.25 Hz (*p* = 0.047), and 2.5 Hz (*p* = 0.038) conditions. Post hoc comparisons also revealed significant difference between the Hastener group and the HOA group for the 1.25 Hz condition (*p* = 0.048). No other comparisons were significant (*p* > 0.140). For movement rate CV, results revealed no significant main effects of size or group and no interaction effect across all pacing rates (Size: *F*(1) < 1.575, *p* > 0.219; Group: *F*(2) < .0.339, *p* > 0.715; Interaction: *F*(2) < 1.444, *p* > 0.252).

**Fig 3:**
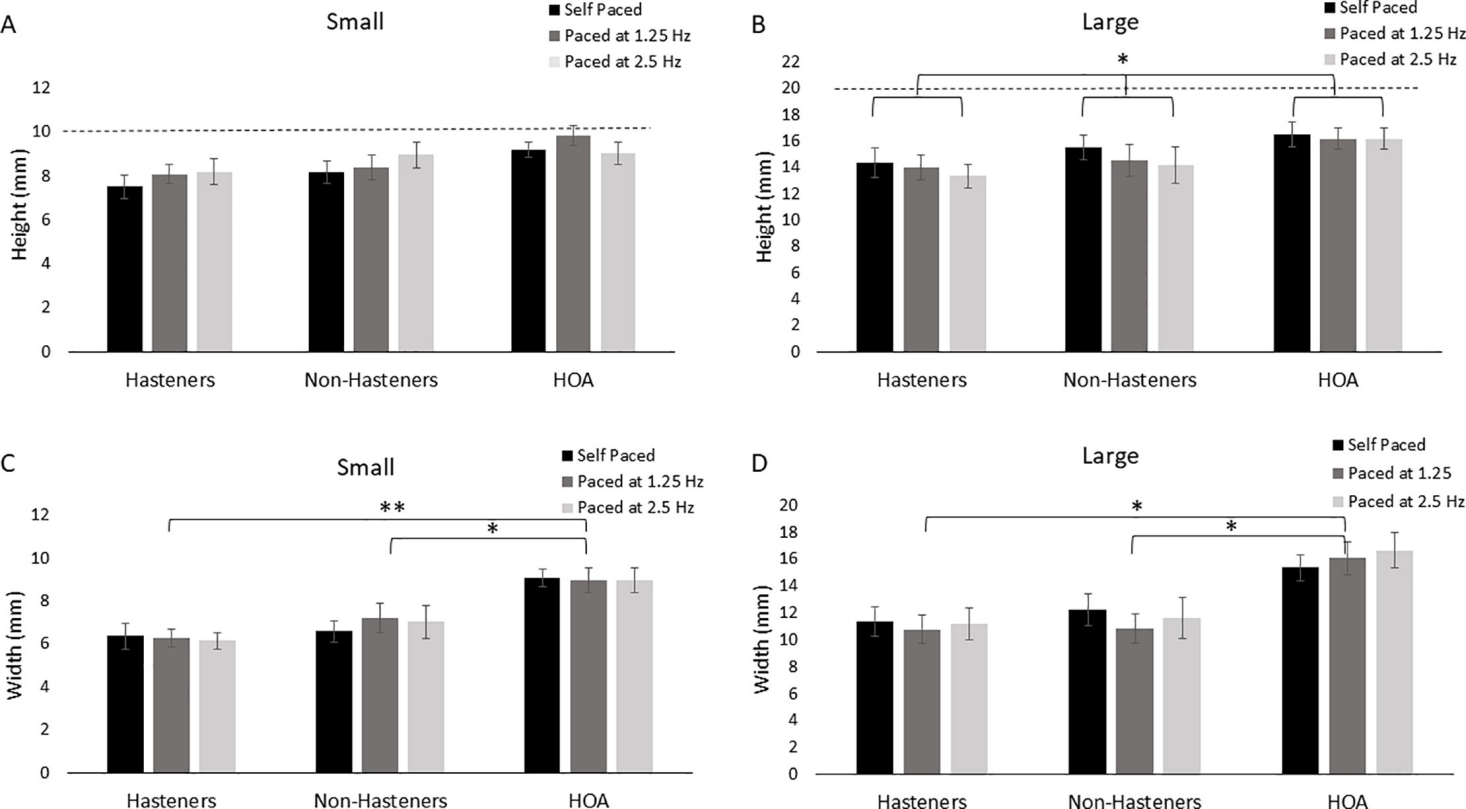
Circle height for all three pacing conditions for the PD Hastener, PD Non-Hastener, and HOA groups for the small (A) and large (B) circle sizes. The dotted line represents the intended height. Brackets indicate a significant difference in pacing condition among all groups. Circle width for all three pacing conditions for the PD Hastener, PD Non-Hastener, and HOA groups for the small (C) and large (D) circle sizes. Brackets indicate significant group differences. *p < 0.05, **p < 0.01.

Fig 3A and 3B show the effects of pacing rate on circle height among groups. Across all pacing rates and both circle sizes, height was smaller than the given lined cue (1 cm for small and 2 cm for large) for all groups. In general, the height of the circle increased when provided an auditory pacing cue for small circle, but decreased for large circle. Statistical results revealed a main effect of rate for the large circle only (*F*(1) = 3.252, *p* = 0.045). No main effect of pacing rate was revealed for the small circle (*F*(1) = 1.635, *p* = 0.204), and no main effect of group was revealed for either the small (*F*(2) = 2.316, *p* = 0.116) or large (*F*(2) = 1.383, *p* = 0.266) circles. Moreover, no interaction effects were revealed for either circle size [small: (*F*(2) = 1.146, *p* = 0.343); large: (*F*(2) = 0.471, *p* = 0.756)]. Post hoc results revealed a significant difference in height for large circles between the self-paced and 2.5 Hz conditions only (*p* = 0.015). No other comparisons were significant (*p* > 0.12). For circle height CV, results revealed no significant main effects of pacing rate or group and no interaction effect across both sizes (Rate: *F*(1) < 3.771, *p* > 0.062; Group: *F*(2) < .2.901, *p* > 0.070; Interaction: *F*(2) < 1.241, *p* > 0.304).

**Fig 4:**
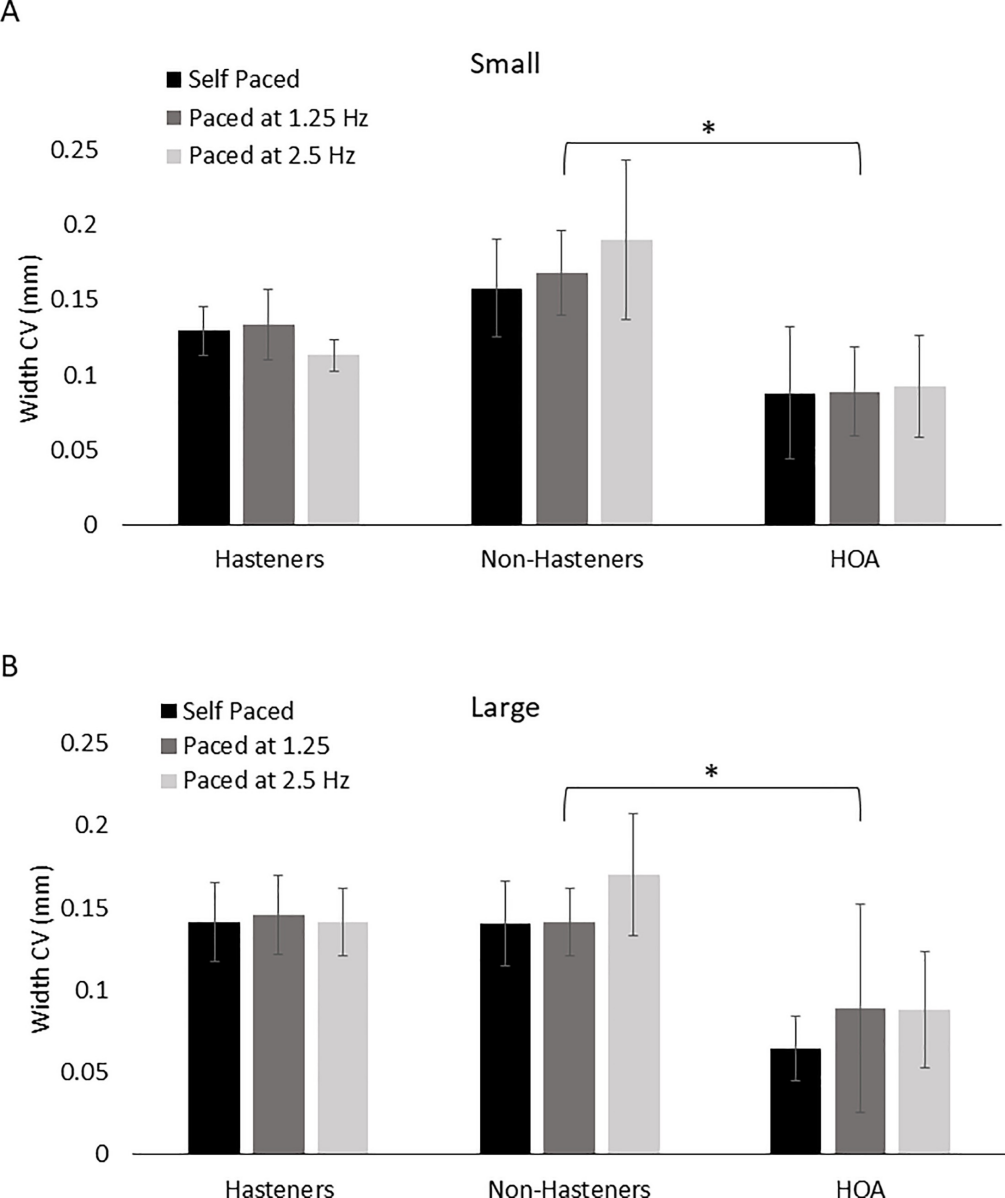
Width coefficient of variation for all three pacing conditions for the PD Hastener, PD Non-Hastener, and HOA groups for the small (A) and large (B) circle sizes. Brackets indicate significant group differences. *p < 0.05.

Fig 3C and 3D show the effects of pacing rate on circle width among groups. Note that no lined cues were provided for width. For circle width, both PD groups (Hasteners and Non-Hasteners) were smaller compared to the HOA group, but no consistent pattern was observed across pacing rate. Statistical results revealed a main effect of group for both the small (*F*(1) = 7.379, *p* = 0.002) and large (*F*(1) = 6.180, *p* = 0.0.006) circles. No main effects of pacing rate (Small: *F*(2) = 0.268, *p* = 0.765; Large: *F*(2) = 1.025, *p* = 0.365) or interaction effects (Small: *F*(2) = 0.379, *p* = 0.823; Large: *F*(2) = 0.1.849, *p* = 0.131) were revealed. Post hoc comparisons revealed significant differences between the Hastener and HOA groups for both small (*p* = 0.003) and large (p = 0.013) circles and between the Non-Hastener and HOA groups for both the small (*p* = 0.015) and large (*p* = 0.013) groups. No other comparisons were significant (*p* > 0.663). For circle width CV, results revealed a significant main effect of group for both the small (*F*(2) = 4.512, *p* = 0.019) and large (*F*(2) = 3.813, *p* = 0.033) circles (Fig 4). No main effect of pacing rate (*F*(1) < 2.702, *p* > 0.111) or interaction (*F*(2) > 1.011, *p* < 0.376) was revealed for either small or large circles. Post hoc results revealed a significant difference in circle width CV between the Non-Hastener and HOA group for both the small (*p* = 0.015) and large (*p* = 0.037) circles. No other comparisons were significant (*p* > 0.107).

Table 2 shows the mean and standard error for all EMG outcome measures. Statistical results for the effect of circle size among groups revealed only one main effect of size for the 1.25 Hz pacing condition for peak FDI amplitude (*F*(1) = 5.687, *p* = 0.025). No other main effects of size or group and no other interaction effects were revealed. Statistical results for the effect of pacing rate among groups revealed a main effect of pacing rate for both the small and large circles for EDC area (Small: *F*(2) = 4.757, *p* = 0.013; Large: *F*(2) = 21.348, *p* < 0.001) and FDI area (Small: *F*(2) = 4.242, *p* = 0.002; Large: *F*(2) = 3.646, *p* = 0.033). No other main effects of size or group and no other interaction effects were revealed. Post hoc results revealed a significant difference in EDC area between the self-paced and 1.25 Hz conditions (*p* = 0.001), the self-paced and 2.5 Hz conditions (*p* < 0.001), and the 1.25 Hz and 2.5 Hz conditions (p < 0.001). For FDI area, significant differences were revealed between the self-paced and 2.5 Hz conditions (*p* = 0.016) and the 1.25 Hz and 2.5 Hz conditions (*p* = 0.015). No other comparisons were significant (*p* > 0.017, Bonferonni corrected).

**Table 2:** Mean and standard error for electromyography outcome measures.

Peak EDC Amplitude (V)						
	***Small Self-Paced***	***Small 1*.*25 Hz***	***Small 2*.*5 Hz***	***Large Self-Paced***	***Large 1*.*25 Hz***	***Large 2*.*5 Hz***
**Hasteners**	0.007 ± 0.001	0.007 ± 0.001	0.008 ± 0.001	0.007 ± 0.002	0.007 ± 0.001	0.007 ± 0.001
**Non-Hasteners**	0.007 ± 0.001	0.006 ± 0.001	0.007 ± 0.001	0.006 ± 0.002	0.007 ± 0.001	0.007 ± 0.002
**HOAs**	0.007 ± 0.003	0.006 ± 0.002	0.006 ± 0.001	0.007 ± 0.002	0.007 ± 0.002	0.008 ± 0.003
**Peak FDI Amplitude (V)**						
	***Small Self-Paced***	***Small 1*.*25 Hz***	***Small 2*.*5 Hz***	***Large Self-Paced***	***Large 1*.*25 Hz***	***Large 2*.*5 Hz***
**Hasteners**	0.053 ± 0.029	0.039 ± 0.016	0.049 ± 0.021	0.072 ± 0.031	0.070 ± 0.030	0.062 ± 0.028
**Non-Hasteners**	0.033 ± 0.014	0.047 ± 0.021	0.036 ± 0.016	0.038 ± 0.017	0.062 ± 0.029	0.034 ± 0.013
**HOAs**	0.029 ± 0.009	0.019 ± 0.006	0.017 ± 0.005	0.069 ± 0.040	0.027 ± 0.009	0.025 ± 0.006
**EDC Area (V)**						
	***Small Self-Paced***	***Small 1*.*25 Hz***	***Small 2*.*5 Hz***	***Large Self-Paced***	***Large 1*.*25 Hz***	***Large 2*.*5 Hz***
**Hasteners**	0.003 ± 0.001	0.002 ± 0.0004	0.001 ± 0.0003	0.004 ± 0.001	0.002 ± 0.0004	0.001 ± 0.0002
**Non-Hasteners**	0.003 ± 0.0005	0.002 ± 0.0003	0.002 ± 0.001	0.005 ± 0.001	0.003 ± 0.0004	0.002 ± 0.0003
**HOAs**	0.003 ± 0.001	0.002 ± 0.001	0.001 ± 0.0003	0.003 ± 0.001	0.003 ± 0.001	0.002 ± 0.001
**FDI Area (V)**						
	***Small Self-Paced***	***Small 1*.*25 Hz***	***Small 2*.*5 Hz***	***Large Self-Paced***	***Large 1*.*25 Hz***	***Large 2*.*5 Hz***
**Hasteners**	0.012 ± 0.005	0.011 ± 0.005	0.009 ± 0.004	0.014 ± 0.004	0.015 ± 0.006	0.010 ± 0.005
**Non-Hasteners**	0.014 ± 0.008	0.011 ± 0.004	0.009 ± 0.004	0.020 ± 0.009	0.019 ± 0.010	0.007 ± 0.003
**HOAs**	0.014 ± 0.005	0.008 ± 0.003	0.004 ± 0.001	0.032 ± 0.016	0.012 ± 0.005	0.007 ± 0.002

EDC = Extensor Digitorum Communis; FDI = First Dorsal Interosseous; Hz = Hertz; HOAs = Healthy Older Adults. For Peak FDI Amplitude, shading represents a significant main effect of size. For EDC area and FDI area, shading represents a significant main effect of pacing rate for small (darker shading) and large (lighter shading) circles.

Table 3 shows the mean and standard error for all EMG CV outcome measures. Statistical results for the effect of circle size among groups revealed a main effect of group for the self-paced condition (*F*(2) = 5.911, *p* = 0.008) and a main effect of size for the 1.25 Hz pacing condition (*F*(1) = 3.408, *p* = 0.007), both for peak EDC amplitude. A significant interaction effect for peak EDC amplitude for the 1.25 Hz condition was also revealed (*F*(2) = 6.860, *p* = 0.004). No other main effects or interaction effects were revealed. Post hoc results for the self-paced group comparisons revealed significant differences between the Hastener and HOA groups (*p* = 0.027) and between the Non-Hastener and HOA groups (*p* = 0.014). There was no significant difference between the Hastener and Non-Hastener groups (*p* = 0.993). Statistical results for the effect of pacing rate among groups revealed a main effect of pacing rate for the small circles for peak EDC amplitude (*F*(1) = 5.262, *p* = 0.031) and a main effect of group for the large circle for peak EDC amplitude (*F*(2) = 7.636, *p* = 0.002). An interaction effect was revealed for the small circle for peak FDI amplitude (*F*(2) = 5.112, *p* = 0.014), EDC area (*F*(2) = 8.164, *p* = 0.002), and FDI area (*F*(2) = 8.094, *p* = 0.002). No other main effects of pacing rate or group and no other interaction effects were revealed. Post hoc results for the large circle group comparisons revealed a significant difference between the Hastener and HOA groups (p = 0.023) and the Non-Hastener and HOA groups (p = 0.003). Although there was a main effect of pacing rate for the small circle, there were no significant differences in peak EDC amplitude between rates (*p* > 0.04, Bonferonni corrected).

**Table 3:** Mean and standard error for electromyography coefficient of variation outcome measures.

**Peak EDC Amplitude (V)**						
	***Small Self-Paced***	***Small 1*.*25 Hz***	***Small 2*.*5 Hz***	***Large Self-Paced***	***Large 1*.*25 Hz***	***Large 2*.*5 Hz***
**Hasteners**	0.145 ± 0.019	0.222 ± 0.052	0.193 ± 0.039	0.176 ± 0.024	0.186 ± 0.020	0.192 ± 0.018
**Non-Hasteners**	0.164 ± 0.017	0.159 ± 0.009	0.165 ± 0.016	0.136 ± 0.010	0.161 ± 0.014	0.184 ± 0.015
**HOAs**	0.195 ± 0.011	0.158 ± 0.020	0.266 ± 0.046	0.251 ± 0.034	0.321 ± 0.045	0.267 ± 0.072
**Peak FDI Amplitude (V)**						
	***Small Self-Paced***	***Small 1*.*25 Hz***	***Small 2*.*5 Hz***	***Large Self-Paced***	***Large 1*.*25 Hz***	***Large 2*.*5 Hz***
**Hasteners**	0.481 ± 0.210	0.516 ± 0.231	0.211 ± 0.052	0.496 ± 0.156	0.404 ± 0.078	0.294 ± 0.041
**Non-Hasteners**	0.255 ± 0.046	0.304 ± 0.058	0.0245 ± 0.050	0.312 ± 0.087	0.245 ± 0.033	0.227 ± 0.039
**HOAs**	0.259 ± 0.040	0.235 ± 0.041	0.398 ± 0.065	0.266 ± 0.037	0.359 ± 0.057	0.319 ± 0.067
**EDC Area (V)**						
	***Small Self-Paced***	***Small 1*.*25 Hz***	***Small 2*.*5 Hz***	***Large Self-Paced***	***Large 1*.*25 Hz***	***Large 2*.*5 Hz***
**Hasteners**	0.320 ± 0.094	0.468 ± 0.297	0.141 ± 0.025	0.302 ± 0.076	0.213 ± 0.068	0.174 ± 0.026
**Non-Hasteners**	0.214 ± 0.040	0.1200 ± 0.029	0.189 ± 0.021	0.220 ± 0.041	0.197 ± 0.031	0.195 ± 0.031
**HOAs**	0.184 ± 0.021	0.171 ± 0.022	0.295 ± 0.039	0.269 ± 0.042	0.275 ± 0.033	0.222 ± 0.094
**FDI Area (V)**						
	***Small Self-Paced***	***Small 1*.*25 Hz***	***Small 2*.*5 Hz***	***Large Self-Paced***	***Large 1*.*25 Hz***	***Large 2*.*5 Hz***
**Hasteners**	0.424 ± 0.120	0.721 ± 0.381	0.230 ± 0.045	0.347 ± 0.095	0.348 ± 0.059	0.291 ± 0.026
**Non-Hasteners**	0.224 ± 0.040	0.323 ± 0.076	0.224 ± 0.037	0.257 ± 0.046	0.220 ± 0.029	0.246 ± 0.044
**HOAs**	0.246 ± 0.031	0.208 ± 0.035	0.369 ± 0.054	0.282 ± 0.044	0.310 ± 0.060	0.277 ± 0.079

EDC = Extensor Digitorum Communis; FDI = First Dorsal Interosseous; Hz = Hertz; HOAs = Healthy Older Adults. Dark shaded cells represent a significant main effect of size. Lighter shaded cells represent a significant main effect of group.

## Discussion

The purpose of this study was to compare circle drawing performance in persons with PD who demonstrated impairment in repetitive finger movement and those who did not. The results suggest that, in general, persons with PD demonstrate differences in circle drawing and associated EMG activity compared to HOAs. Circle width and peak EDC CV were significantly smaller for both PD groups compared to HOAs. However, there were some differences between the PD Hastener and PD Non-Hastener groups. Results revealed that the PD Hastener group had a significantly faster movement rate when writing circles (across both circle sizes and all pacing rates) compared to the PD Non-Hastener and HOA groups. However, circle width CV (both small and large circles) was significantly larger in the PD Non-Hastener group compared to the HOA group. Thus, these results suggest that differences in repetitive finger tapping performance in persons with PD may also translate to differences in circle drawing performance.

Previous research has shown that when persons with PD are asked to write under increased processing demand, such as dual task or at a fast pace, performance deteriorates [5,12,13,16]. Specifically, persons with PD tend to undershoot the target size, suggesting that amplitude of movement (i.e. height) is most affected by increased processing demand [5,12]. In this study, all participants undershot the target size, and there were no differences in circle height among groups. In general, participants undershot the larger circle size to a greater extent than the small circle size (~17% for small circles; ~28% for large circles). However, circle height was significantly smaller in the 2.5 Hz condition compared to the self-paced condition. This suggests that both a large target size and a fast pacing rate may increase processing demand resulting in undershooting circle height. In this study, there were no differences in circle height between persons with PD and HOAs. The lack of difference among groups in our results may be due to the presence of lined paper that provided a visual cue for circle height. Previous research has shown that visual cues, lined paper, improve handwriting at sizes similar to those used in this study [17–19]. Taken together, visual cues may improve circle writing tasks, but target size and pacing rate may be factors to consider when evaluating performance on writing tasks.

In contrast to circle height, there was no visual cue provided for circle width. Both PD groups had a significantly smaller circle width compared to HOAs, which may be expected as visual cues improve performance as discussed previously. However, perhaps a more interesting result from this study is that the PD Non-Hastener group demonstrated more variability in circle width than the HOA group. Upon inspection of the data (see Fig 1), participants in the PD Non-Hastener group tended to have a preference to produce right-tilted circle shapes, especially for the large circle, while those in the PD Hastener group did not. There was no measure circle shape specifically in this study, but the increase in width variability in the PD Non-Hastener group may be a result of this shape difference. Previous research has shown similar findings in persons with PD and suggests that the right-tilted shape may be due to a deficiency in wrist and finger coordination [1]. However, the PD Hastener group did not demonstrate an increase in width variability or evidence of right-tilted circle drawing. Additional research is needed to determine the underlying cause of differences in circle drawing performance between the PD Hastener and PD Non-Hastener groups.

Participants in the Hastener group moved significantly faster than participants in the Non-Hastener and HOA groups regardless of circle size and pacing rate. The largest difference in movement rate between the PD groups was observed during the self-paced condition. There was approximately a 1.0 Hz difference in movement rate in the self-paced condition compared to approximately a 0.5 Hz difference in both the 1.25 Hz and 2.5 pacing conditions. This may suggest than an auditory cue may improve timing regulation to some extent in those participants who hasten, but not fully. Performance between PD groups also differed between the two pacing conditions of 1.25 Hz and 2.5 Hz. For the 1.25 Hz pacing condition, both PD groups moved faster than the intended tone. In contrast, for the 2.5 Hz pacing condition, the PD Hastener group moved faster that the intended tone, while the PD Non-Hastener group moved slower than the tone. This observation may be due to the simple fact that the PD Hastener group moved on average 0.5 Hz faster than the PD Non-Hastener group regardless of the pacing rate.

Previous behavioral and electrophysiological research has shown evidence that control of fine motor movement changes near movement rates of 2.0 Hz [20–23]. This change in control is thought to represent the change from discrete to continuous movement and is associated with changes in beta band oscillations recorded over the sensorimotor cortex [20]. Research has shown an association between abnormal beta band oscillations and impairments in repetitive finger movement performance at rates near to and above 2.0 Hz in persons with PD [22,23]. In repetitive finger movement, 2.0 Hz is approximate the rate in which movement performance switched from discrete to continuous movement. Moreover, circle drawing may be considered a continuous movement. Thus, persons with PD that demonstrate hastening at rates near to and above 2.0 Hz (continuous movement) may have abnormal control of other continuous movement that is evident in other fine motor tasks, including circle drawing. Indeed, the behavioral results of this study may support this notion, but future research using additional brain imaging techniques during writing tasks in persons with PD are needed.

### Limitations

All participants with PD in this study were tested in their optimal medicated state. While it has been shown that medication does not improve repetitive finger movement performance at rates near to and above 2.0 Hz [8], medication has been shown to improve handwriting in persons with PD [4]. Continued research is needed to examine the effects of medication on handwriting in PD Hasteners and PD Non-Hasteners. In addition, not all participants used the more affect side to complete the drawing task. Thus, in some participants, finger tapping and circle drawing were done with different hands. However, there were no differences between PD groups on the percentage of participants with the dominant hand as the most affected side. Nonetheless, this study provides evidence for future research aimed at understanding fine motor control in persons with PD.

### Conclusion

This study revealed that persons with PD that demonstrate impairments in repetitive finger movements, specifically hastening, also demonstrate increased movement rate during circle drawing, while those persons with PD that do not hasten demonstrate impairments in width variability. This suggests that there may be differing impairments across individuals with PD when writing quickly. Further research is needed to better understand differences in writing performance among persons with PD so that more patient-centered treatments for writing, and potentially other fine motor skills, can be developed.

## Supporting information

S1 Table Mean and standard error for kinematic variables. (DOCX)

S2 Table Mean and standard error for kinematic coefficient of variation variables. (DOCX)
