# Supplementary material for: Repetitive finger movement and circle drawing in persons with Parkinson’s disease
Source: PLoS One. 2019 Sep 23;14(9):e0222862. doi: 10.1371/journal.pone.0222862 (PMC6756750; doi:10.1371/journal.pone.0222862)
Supplement: S1 Table — (DOCX) [file pone.0222862.s001.docx]

**Supplemental Table 1.** Mean and Standard Error for Kinematic Variables

| **Movement Rate (Hz)** | | | | | | |
| --- | --- | --- | --- | --- | --- | --- |
|  | ***Small Self-Paced*** | ***Small 1.25 Hz*** | ***Small 2.5 Hz*** | ***Large Self-Paced*** | ***Large 1.25 Hz*** | ***Large 2.5 Hz*** |
| **Hasteners** | 1.99 ± 0.33 | 2.04 ± 0.20 | 2.96 ± 0.21 | 1.51 ± 0.34 | 1.99 ± 0.25 | 2.68 ± 0.10 |
| **Non-Hasteners** | 0.98 ± 0.14 | 1.62 ± 0.10 | 2.41 ± 0.16 | 0.70 ± 0.07 | 1.48 ± 0.09 | 2.17 ± 0.18 |
| **HOAs** | 1.39 ± 0.24 | 1.59 ± 0.04 | 2.77 ± 0.17 | 1.36 ± 0.15 | 1.48 ± 0.15 | 2.51 ± 0.07 |
| **Circle Height (mm)** | | | | | | |
|  | ***Small Self-Paced*** | ***Small 1.25 Hz*** | ***Small 2.5 Hz*** | ***Large Self-Paced*** | ***Large 1.25 Hz*** | ***Large 2.5 Hz*** |
| **Hasteners** | 7.50 ± 0.52 | 8.09 ± 0.42 | 8.18 ± 0.59 | 14.36 ± 1.11 | 14.01 ± 0.94 | 13.33 ± 0.92 |
| **Non-Hasteners** | 8.17 ± 0.51 | 8.39 ± 0.58 | 8.96 ± 0.61 | 15.53 ± 0.93 | 14.52 ± 1.19 | 14.15 ± 1.37 |
| **HOAs** | 9.18 ± 0.36 | 9.84 ± 0.47 | 9.04 ± 0.51 | 16.50 ± 0.92 | 16.19 ± 0.81 | 16.18 ± 0.84 |
| **Circle Width (mm)** | | | | | | |
|  | ***Small Self-Paced*** | ***Small 1.25 Hz*** | ***Small 2.5 Hz*** | ***Large Self-Paced*** | ***Large 1.25 Hz*** | ***Large 2.5 Hz*** |
| **Hasteners** | 6.38 ± 0.60 | 6.28 ± 0.44 | 6.15 ± 0.37 | 11.34 ± 1.09 | 10.78 ± 1.03 | 11.19 ± 1.17 |
| **Non-Hasteners** | 6.59 ± 0.49 | 7.19 ± 0.68 | 7.03 ± 0.79 | 12.22 ± 1.17 | 10.82 ± 1.12 | 11.62 ± 1.52 |
| **HOAs** | 9.09 ± 0.42 | 8.96 ± 0.58 | 8.98 ± 0.56 | 15.38 ± 0.97 | 16.07 ± 1.24 | 16.65 ± 1.32 |

Hz = Hertz; HOAs = Healthy Older Adults; mm = millimeters
