# Supplementary material for: Repetitive finger movement and circle drawing in persons with Parkinson’s disease
Source: PLoS One. 2019 Sep 23;14(9):e0222862. doi: 10.1371/journal.pone.0222862 (PMC6756750; doi:10.1371/journal.pone.0222862)
Supplement: S2 Table — (DOCX) [file pone.0222862.s002.docx]

**Supplemental Table 2.** Mean and Standard Error for Kinematic Coefficient of Variation Variables

| **Movement Rate (Hz)** | | | | | | |
| --- | --- | --- | --- | --- | --- | --- |
|  | ***Small Self-Paced*** | ***Small 1.25 Hz*** | ***Small 2.5 Hz*** | ***Large Self-Paced*** | ***Large 1.25 Hz*** | ***Large 2.5 Hz*** |
| **Hasteners** | 0.21 ± 0.21 | 0.17 ± 0.03 | 0.17 ± 0.03 | 0.21 ± 0.03 | 0.30 ± 0.14 | 0.18 ± 0.14 |
| **Non-Hasteners** | 0.19 ± 0.03 | 0.20 ± 0.04 | 0.18 ± 0.03 | 0.19 ± 0.04 | 0.18 ± 0.03 | 0.18 ± 0.02 |
| **HOAs** | 0.22 ± 0.10 | 0.16 ± 0.03 | 0.19 ± 0.05 | 0.11 ± 0.01 | 0.23 ± 0.06 | 0.13 ± 0.01 |
| **Circle Height (mm)** | | | | | | |
|  | ***Small Self-Paced*** | ***Small 1.25 Hz*** | ***Small 2.5 Hz*** | ***Large Self-Paced*** | ***Large 1.25 Hz*** | ***Large 2.5 Hz*** |
| **Hasteners** | 0.11 ± 0.02 | 0.12 ± 0.01 | 0.11 ± 0.01 | 0.12 ± 0.02 | 0.13 ± 0.03 | 0.11 ± 0.02 |
| **Non-Hasteners** | 0.11 ± 0.01 | 0.19 ± 0.08 | 0.07 ± 0.01 | 0.10 ± 0.01 | 0.10 ± 0.02 | 0.11 ± 0.02 |
| **HOAs** | 0.06 ± 0.004 | 0.07 ± 0.01 | 0.09 ± 0.01 | 0.05 ± 0.01 | 0.07 ± 0.01 | 0.08 ± 0.01 |
| **Circle Width (mm)** | | | | | | |
|  | ***Small Self-Paced*** | ***Small 1.25 Hz*** | ***Small 2.5 Hz*** | ***Large Self-Paced*** | ***Large 1.25 Hz*** | ***Large 2.5 Hz*** |
| **Hasteners** | 0.13 ± 0.02 | 0.13 ± 0.02 | 0.11 ± 0.01 | 0.17 ± 0.02 | 0.15 ± 0.02 | 0.14 ± 0.02 |
| **Non-Hasteners** | 0.16 ± 0.03 | 0.17 ± 0.03 | 0.19± 0.05 | 0.14 ± 0.03 | 0.14 ± 0.02 | 0.17 ± 0.04 |
| **HOAs** | 0.09 ± 0.01 | 0.09 ± 0.01 | 0.09 ± 0.01 | 0.06 ± 0.01 | 0.09 ± 0.02 | 0.09 ± 0.01 |

Hz = Hertz; HOAs = Healthy Older Adults; mm = millimeters
